# Lacking Clarity or Strategic Ambiguity? Comment on "Competing Frames in Global Health Governance: An Analysis of Stakeholder Influence on the Political Declaration on Non-communicable Diseases"

**DOI:** 10.34172/ijhpm.2021.113

**Published:** 2021-09-01

**Authors:** Angela Carriedo, Kathrin Lauber, Margaret M. Miller, Rob Ralston

**Affiliations:** ^1^World Public Health Nutrition Association, London, UK.; ^2^Tobacco Control Research Group, Department for Health, University of Bath, Bath, UK.; ^3^School of Medical and Health Sciences, Edith Cowan University, Perth, WA, Australia.; ^4^Global Health Policy Unit, Social Policy, School of Social and Political Science, University of Edinburgh, Edinburgh, UK.

**Keywords:** Multi-stakeholder Partnerships, Ambiguity, Global Health Governance, Conflicts of Interests, Unhealthy Commodity Industries

## Abstract

This commentary engages with Suzuki and colleagues’ analysis about the ambiguity of multi-stakeholder discourses in the United Nations (UN) Political Declaration of the 3rd High-Level Meeting of the General Assembly on the Prevention and Control of Non-Communicable Diseases (HLM-NCDs), suggesting that blurring between public and private sector in this declaration reflects broader debates about multi-stakeholder partnerships (MSPs) and public-private partnerships (PPPs) in health governance. We argue that the ambiguity between the roles and responsibilities of public and private actors involved may downplay the role (and regulation) of conflicts of interest (COI) between unhealthy commodity industries and public health. We argue that this ambiguity is not simply an artefact of the Political Declaration process, but a feature of multi-stakeholderism, which assumes that commercial actors´ interests can be aligned with the public interest. To safeguard global health governance, we recommend further empirical and conceptual research on COI and how it can be managed.

 The increasing prominence of multi-stakeholder fora and partnerships in global health^1-4^ raises questions about how to identify and manage potential tensions between private sector and public heath interests. In their analysis of stakeholder influence on the United Nations (UN) Political Declaration of the 3^rd^ High-Level Meeting of the General Assembly on the Prevention and Control of Non-Communicable Diseases (HLM-NCDs), Suzuki et al highlight how the drafting of the Political Declaration was marked by ambiguity and confusion about the participation of non-state actors in health governance. Their findings suggest that the involvement of the private sector in multi-stakeholder consultations is likely to frustrate efforts to develop coherent political commitments to addressing the structural drivers of NCDs. In this commentary, we argue that the ambiguity identified by Suzuki et al in their analysis of the interactive consultations over the UN HLM-NCDs is a defining and strategic feature of multi-stakeholder discourses. Ambiguity blurs the contrast between public and private sectors, and minimises potential tensions between commercial interests and public health interests.

 While Suzuki et al make an analytical distinction between ‘whole-of-government’ and ‘whole-of-society’ approaches to health governance, we suggest that multi-stakeholder discourses blur the distinction between state, civil society and private sector actors; and in the process draw attention away from private sector influence in health governance. In this commentary, we argue that ambiguity may be strategically used to downplay the identification and management of conflicts of interest (COI) within collaborative modes of health governance.

 When engaging with the private sector, particularly unhealthy commodity industries (eg, alcohol, ultra-processed foods and sugar-sweetened beverages, industrial agribusiness) – hereafter commercial actors –, multilateral organisations should consider the potential risks of such interactions for global health governance. These commercial actors have consistently used similar policy frames to push for their economic interests that rarely align with health-related outcomes.^4-6^ We need to better understand how public health mandates are discussed and decided upon, and how policies are developed to ensure transparency and equity in such governance arrangements, where non-state actors interact at different jurisdictional levels.^4,7^

## Collaboration and Partnership: The Logic of Multi-stakeholderism

 In reviewing the UN HLM, Suzuki et al make a conceptual distinction between ‘whole-of-government’ approaches to public health – which they describe as coordination across government agencies –, and differentiate this from ‘whole-of-society’ or ‘multi-stakeholder’ governance in which non-state actors (including the private sector) are consulted over the development of policy instruments and solutions to address public health issues. However, while the authors are clear about the distinction between state-centred and multi-stakeholder approaches, they note that the language of the UN HLM Political Declaration blurs the contrast between non-state actors.

 While supporting this distinction, we would argue that what Suzuki and colleagues’ identify as the ‘interchangeability’ of these terms in the UN HLM-NCDs Declaration symbolises a wide-ranging commitment to multi-stakeholder partnership (MSP) and public-private partnerships (PPPs) as key mechanisms to achieving health and development goals. In this sense, the UN HLM-NCDs Declaration reflects a broader logic of multi-stakeholder inclusivity, modelled on policy deliberation and dialogue between state and non-state actors.^8^ Importantly, this logic reinforces the idea that engagement processes should involve the private sector (including commercial actors) as a core stakeholder in the design and implementation of policy. The implication for health governance is that important differences interests and motivations between civil society and commercial actors engagement are minimised and collapsed within MSP and PPPs. Building on Suzuki and colleagues’ analysis, we would suggest that this observed ambiguity in the roles, responsibilities and mandates of non-state actors is not simply an artefact of the UN HLM-NCDs process, but a feature of multi-stakeholderism in which it is assumed that commercial actors´ interests can be aligned with the public interest.

## Marginalising Conflict of Interest

 In addition to the ambiguity between types of stakeholder involved in the HLM hearings, Suzuki et al note how identifying and managing COIs between commercial and public health interests was not incorporated in the final document, despite ‘strong advocacy from non-governmental organizations (NGOs), academic institutions and some Member States.’ This concern was articulated by one civil society organisation, which noted that ‘the declaration encourages multi-sectoral partnerships, but the risks associated with COIs are mentioned only briefly.’ With this outcome, the Declaration illustrates that the perception of COI is peripheral to health governance; and it is illustrative of wider fault-lines in global health. As the authors argue, the promotion of MSP and PPPs in the absence of tools to manage COI, ‘is the worst possible combination from the perspective of many NGOs and public health advocates.’ We would argue that the elastic, malleable qualities of multi-stakeholderism can be purposefully constructed to downplay the significance of COI in the process of policy-making.^9^

 Suzuki and colleagues’ findings on the issue of COI are particularly worrying. This resonates with previous work on this topic showing that in other public consultations, commercial actors saw the topic as redundant and unnecessary^6^ and it was ‘effectively addressed’ by individual disclosures; and that such guidelines will limit the knowledge, expertise, and resources the private sector can contribute to public health programmes.^6^ Nevertheless, NGOs and Member States have shown support for UN tools developed to address COI. ^7^

 Two issues arise from the evidence now available: (*a*) the implications of not having an unambiguous understanding of what a COI is; and (*b*) the issues arising from the mischaracterisation of COI as a peripheral concern which can be straightforwardly managed through disclosure.

 First, the divergence of understandings of COI suggests that clarity is urgently needed.^10^ In the sphere of public health governance, experts should aim to build a consensus for a particular reason: the actors involved will intrinsically have divergent interests, but we need a clear understanding of when and how interactions enable a convergence on a primary interest, without being jeopardised by any other competing interest; and more importantly who should be included in consultative processes for global health policy-making.^9^ One way forward to reach clarity on the concepts of COI and ‘commercial influence’ could be through concept mapping,^11^ an approach which enables the collection of various actors’ ideas to produce a structured conceptual framework, similar to previous exercises on integrity principles in population health.

 Second, disclosure as a mechanism to address COI has been the predominant solution among MSP, PPPs or ‘whole-of-society’ approaches. We agree with the authors and other scholars^12^ that disclosure is not sufficient to address or avoid (institutional) COI or corporate interference in nutrition policy-making when the institutional structure of policy-making is the overarching issue. Nevertheless, ‘whole-of-society’ and ‘multi-stakeholder’ approaches are the dominant model of health governance. We are faced with questions about MSP’s benefits and appropriateness. Multilateral agencies need to urgently clarify and address issues around MSPs and PPPs in public health. Rethinking the terms of engagement with commercial sector actors require posing questions such as: (*a*) Can they be meaningfully involved where their fiduciary duties to shareholders conflict with the values/aims of the initiative/programme at hand? (*b*) What principles or codes of conduct will be implemented? (*c*) What actors will monitor compliance and hold private sector actors to account? (*d*) How will transparency of NGO funding be ensured to mitigate any potential vested or conflicting interests? (*e*) How to integrate into these arrangements the right to health: adequate access to healthy food and a healthy environment? (*f*) How to include the genuine concern of addressing vulnerable populations and achieving related human rights?

 The ‘whole-of-society’ or ‘multi-stakeholder’ approach encompasses, as Suzuki et al mention, cooperation among state and non-state actors, including civil society and the private sector. This raises questions about the extent to which tensions and competing interests can be reconciled in this model of governance, and what tools and design choices can effectively regulate COI in public health policy. Can any potential negative impacts be mitigated and, if so, how? Are some commercial actors better suited for engagement than others? Or should engagement take the same shape as the non-engagement with the tobacco industry under the World Health Organization (WHO) Framework Convention on Tobacco Control?^13^ What do Member States and, more importantly, their citizens gain from such partnerships, and what do they give up? What do partners stand to gain or lose? What processes for transparency and accountability are required and followed?

## Discussion

 Suzuki and colleagues’ work gives us important insights into the tip of the iceberg surrounding structural problems of the ‘multi-stakeholder’ and ‘whole-of-society’ approaches in the global health agenda. Their work resonates with previous research exploring how food industry actors have attempted to shape debates around NCD policy and COI through multi-stakeholder arrangements and PPPs.^4-6^ We argue that the ambiguity of the language in the Political Declaration has been a tool to downplay the significance of wider structural and institutional conflicts between commercial interests and public health. While public consultations by the UN are based on the principles of inclusiveness, plurality, and democracy, they have legitimised the participation of powerful commercial actors whose health-harming interests may shape outcome documents. We suggest that, as it is, the ambiguous framing of multi-stakeholderism reflected in statements from private sector actors as well as the final Declaration, risks negating the importance of COI in favour of collaboration. This observed ambiguity in the roles, responsibilities and mandates of non-state actors is not simply an artefact of the UN HLM process, but a feature of multi-stakeholderism in which it is assumed that commercial actors’ interests can be aligned with the public interest.

 Amidst current trends of privatisation of health, the proliferation of PPPs at national and global levels; and with private financing of multilateral organisations by food, beverage and agribusiness for the ‘food systems transformation,’ such as the WHO Foundation and the Food System Summit, global health governance processes need much more scrutiny than ever before.^3^

 Mechanisms and tools to safeguard the public’s interests have been designed, such as the Framework Convention on Tobacco Control point 5.3 or the draft tool to prevent and manage conflict of interest in nutrition policy under the WHO mandate: but are often absent in relation to other corporations.^7,13^ Responding to Suzuki and colleagues’ question about what should be included in consultation and decision-making over health policy, we would suggest: (*a*) having an independent committee to scrutinise participation, (*b*) due-diligence of civil society participants, as industry funding may constitute a conflict of interest, (*c*) including a human rights approach to health, (*d*) mapping the inclusion and omission of proposed changes and reasons for including/dismissing them. Some of these can enable non-industry participants to gain and maintain a loud voice, regardless of the private sectors’ permanent and powerful participation.

 Unless there continues to be a push for the implementation of clear definitions, tools and actions against corporate influence, and unless evidence on its effectiveness becomes stronger, the agenda on COI risks being overshadowed/overtaken by principles set by existing PPPs,^14^ particularly in the face of constant pressure from the unhealthy commodities to be part of the solution to NCDs and other health issues, like COVID-19.^15^ More positively, several Member States such as Chile, Mexico, South Africa, and Thailand (notably sugar sweetened beverage taxation and food warning labels), highlight that bold public health policies can be advanced, despite pushback from commercial interests. Conversely, the centrality of multi-stakeholderism to the United Nations Food Systems Summit, in which transnational food companies such have played a central role, and widely opposed by civil society groups and schoolars,^3^ illustrates the wider significance and the risks posed by Suzuki et al and further discussed in this commentary.

## Ethical issues

 Not applicable.

## Competing interests

 Authors declare that they have no competing interests.

## Authors’ contributions

 AC and RR contributed to the drafting and revision of the manuscript. KL and MM contributed with revisions of the manuscript.
